# Supplementary material for: Regulation of B cell fate by chronic activity of the IgE B cell receptor
Source: eLife. 2016 Dec 9;5:e21238. doi: 10.7554/eLife.21238 (PMC5207771; doi:10.7554/eLife.21238)
Supplement: Supplementary file 1. — DOI: http://dx.doi.org/10.7554/eLife.21238.016 [file elife-21238-supp1.docx]

**Supplementary file 1. Table of reagents used in flow cytometry.**

| Antibody Target or Reagent Designation | Antibody RRID | Clone | Conjugates | Vendor | Dilution Factor |
| --- | --- | --- | --- | --- | --- |
| Active Caspase-3 | [AB_1727414](http://antibodyregistry.org/AB_1727414) | C92-605 | Alexa Fluor 647 | BD Biosciences | 20 |
| Annexin V | N/A | N/A | Biotin | BD Biosciences | 20 |
| Annexin V | N/A | N/A | APC | BioLegend | 20 |
| B220 (CD45R) | [AB_493717](http://antibodyregistry.org/AB_493717) | RA3-6B2 | Alexa Fluor 700 | BioLegend | 100 |
| B220 (CD45R) | [AB_10374576](http://antibodyregistry.org/AB_10374576) | RA3-6B2 | Qdot 655 | Life Technologies | 250-300 |
| B220 (CD45R) | [AB_10563910](http://antibodyregistry.org/AB_10563910) | RA3-6B2 | V500 | BD Biosciences | 100 |
| CD138 (Syndecan-1) | [AB_394999](http://antibodyregistry.org/AB_394999), [AB_1645216](http://antibodyregistry.org/AB_1645216), [AB_395000](http://antibodyregistry.org/AB_395000) | 281-2 | Biotin, PE, APC, BV711 | BD Biosciences | 100-150 |
| CD16 and CD32 | [AB_1574975](http://antibodyregistry.org/AB_1574975) | 93 | Purified | BioLegend | 50 |
| CD19 | [AB_1645270](http://antibodyregistry.org/AB_1645270) | 1D3 | V450 | BD Biosciences | 100 |
| CD19 | [AB_11218994](http://antibodyregistry.org/AB_11218994) | 6D5 | BV785 | BioLegend | 50 |
| CD38 | [AB_657740](http://antibodyregistry.org/AB_657740) | 90 | Alexa Fluor 700 | eBioscience | 75 |
| CD38 | [AB_2275531](http://antibodyregistry.org/AB_2275531) | 90 | PE-Cy7 | BioLegend | 150 |
| CD45.1 | [AB_492866](http://antibodyregistry.org/AB_492866), [AB_493733](http://antibodyregistry.org/AB_493733) | A20 | Pacific Blue, Alexa Fluor 700 | BioLegend | 75 |
| CD45.2 | [AB_492872](http://antibodyregistry.org/AB_492872) | 104 | Pacific Blue | BioLegend | 100 |
| CD69 | [AB_313107](http://antibodyregistry.org/AB_313107) | H1.2F3 | Biotin | BioLegend | 100 |
| Fixable Viability Dye eFluor 780 | N/A | N/A | N/A | eBioscience | 600 |
| HEL | N/A | N/A | Alexa Fluor 647 | Conjugated in house (see methods) | 600 |
| IgD | N/A | 11-26c | Biotin | Southern Biotechnology | 200 |
| IgD | N/A | 11-26c.2a | BV510 | BD Biosciences | 100 |
| IgD | [AB_893528](http://antibodyregistry.org/AB_893528), [AB_1575113](http://antibodyregistry.org/AB_1575113) | 11-26c.2a | Alexa Fluor 647, PerCP-Cy5.5 | BioLegend | 100-200 |
| IgE | [AB_394850](http://antibodyregistry.org/AB_394850) | R35-118 | Biotin | BD Biosciences | 1000-2000 |
| IgE | [AB_315073](http://antibodyregistry.org/AB_315073) | RME-1 | Purified | BioLegend | 15 |
| IgE | [AB_493289](http://antibodyregistry.org/AB_493289), [AB_493290](http://antibodyregistry.org/AB_493290) | RME-1 | FITC, PE | BioLegend | 300-800 |
| IgG1 | [AB_10894002](http://antibodyregistry.org/AB_10894002), [AB_393553](http://antibodyregistry.org/AB_393553),  [AB_394862](http://antibodyregistry.org/AB_394862), [AB_1645625](http://antibodyregistry.org/AB_1645625) | A85-1 | FITC, V450, PE, APC | BD Biosciences | 200-500 |
| Igλ | [AB_345332](http://antibodyregistry.org/AB_345332) | RML-42 | Biotin | BioLegend | 400 |
| Igκ | [AB_1727536](http://antibodyregistry.org/AB_1727536) | 187.1 | PerCP-Cy5.5 | BD Biosciences | 200 |
| IgM | [AB_465939](http://antibodyregistry.org/AB_465939),  [AB_469655](http://antibodyregistry.org/AB_469655) | II/41 | PE, PE-Cy7 | eBioscience | 300 |
| NP | N/A | N/A | APC | Conjugated in house (see methods) | 200-6000 |
| PNA | N/A | N/A | FITC, Biotin | Vector Laboratories | 500, 2000 |
| Streptavidin | N/A | N/A | Qdot 605 | Life Technologies | 400 |
| Streptavidin | N/A | N/A | BV605 | BD Biosciences | 100 |
| Y-Ae | [AB_657822](http://antibodyregistry.org/AB_657822) | EbioY-Ae | Biotin | eBioscience | 500 |
